# From cars to bikes – The effect of an intervention providing access to different bike types: A randomized controlled trial

**DOI:** 10.1371/journal.pone.0219304

**Published:** 2019-07-10

**Authors:** Helga Birgit Bjørnarå, Sveinung Berntsen, Saskia J te Velde, Aslak Fyhri, Benedicte Deforche, Lars Bo Andersen, Elling Bere

**Affiliations:** 1 Department of Public Health, Sport and Nutrition, Faculty of Health and Sport Sciences, University of Agder, Kristiansand, Norway; 2 Department of Safety and the Environment, Institute of Transport Economics, Oslo, Norway; 3 Department of Public Health and Primary Care, Faculty of Medicine and Health Sciences, Ghent University, Ghent, Belgium; 4 Physical Activity, Nutrition and Health Research Unit, Faculty of Physical Education and Physical Therapy, Vrije Universiteit Brussel, Brussels, Belgium; 5 Western Norwegian University of Applied Sciences, Faculty Education, Arts and Sports, Sogndal Campus, Sogndal, Norway; 6 Department of Health and Inequalities, & Centre for Evaluation of Public Health Measures Norwegian Institute of Public Health, Oslo, Norway; University of British Columbia, CANADA

## Abstract

**Introduction:**

We aimed to investigate whether providing parents with children in kindergarten with access to different bicycle types could influence (i) travel behavior and cycling amount, and (ii) intrinsic motivation for cycling and psychological constructs related to car use.

**Methods:**

A randomized, controlled trial was conducted in Southern Norway from September 2017 to June 2018. In total 36 parents were recruited and randomly drawn into an intervention (*n* = 18) or control group (*n* = 18). The intervention group was in random order equipped with an e-bike with trailer (*n* = 6), a cargo (longtail) bike (*n* = 6) and a traditional bike with trailer (*n* = 6).

**Results:**

At follow-up, more participants from the intervention group (vs. the control group) were classified as cyclists to the workplace (*n* = 7 (38.9%) vs. *n* = 1 (5.9%), *p* = 0.04), but not to the kindergarten (*n* = 6 (33.3%) vs. *n* = 2 (11.8%), *p* = 0.23) or to the grocery store (*n* = 2 (11.1%) vs. *n* = 0 (0%), *p* = 0.49). A significant (*p* = ≤0.05) increase in cycling frequency (0.1 to 2.0 days/week) from baseline to follow-up was found in the intervention group for all destinations and seasons, except to the grocery store during winter (*p* = 0.16). A decrease in frequency of car driving (-0.2 to -1.7 days/week) was found to be apparent in terms of travelling to the workplace and the kindergarten for all seasons, yet not to the grocery store for any season (*p* = 0.15–0.49). The intervention group (vs. the control group) reported significantly higher “intrinsic regulation” for cycling (*p* = 0.01) at follow-up.

**Conclusion:**

Access to different bike types for parents with children attending kindergarten resulted in overall increased cycling, decreased car use and higher intrinsic motivation for cycling. E-bikes obtained the greatest cycling amount in total, with the smallest sample variability. Hence, providing parents with children in kindergarten with access to e-bikes might result in increased and sustained cycling, also during the winter season.

## Introduction

Most adults are insufficiently physically active [1, 2], i.e. below the recommended 150 minutes of moderate-to-vigorous intensity physical activity (MVPA) per week [3, 4]. Cycling for transport may be a time-efficient way to integrate physical activity (PA) into daily routines, possibly increasing total PA levels [5, 6]. As transport cycling commonly reaches moderate intensity [7], enhanced cycling could prevent non-communicable diseases and decrease mortality [8–10]. When accounting for inhaled air pollution and traffic accidents, the health benefits for individuals shifting from car to bike substantially outweigh the risks [11], with large societal benefits as well [12, 13]. In addition, replacing motorized modes of transport with everyday cycling has positive environmental effects, through a decline in greenhouse gas emissions [12, 14], noise and pollution [6].

Electric assisted bicycles (e-bikes) have emerged as a new mode of transportation, potentially health enhancing, which is attributable to overall increased levels of PA [15, 16]. Although entailing lower intensity than traditional bikes, e-bikes are found to attain the prescribed MVPA-standard in both active and inactive individuals, as well as in healthy adults and patient groups [17]. E-bikes enable speed maintenance with less effort, thus overcoming typical barriers to traditional pedal cycling like poor fitness, lengthy distances, hilly terrain, lack of time, and lack of end trip facilities (e.g. changing rooms and showers) [18]. Consequently, e-bike users tend to cycle more often and over longer distances [18–21].

Cargo bikes might provide benefits in terms of the transport of equipment and children due to substantial carrying capacity [22]. Because of the weight, it is likely that human-powered cargo bikes imply a higher PA intensity than traditional bikes, which in turn could generate additional health effects [23]. American data report a decline in car travel among cargo bike owners [22], illustrating the car-substitution potential. Although men are repeatedly reported to cycle more than women [24], recent Swiss data indicates that males and females find cargo bikes equally appealing [25]. Besides, if conditions and circumstances are favorable, the cargo bike has been shown to function as a substitute for the car for American mothers with access to one [26]. Nonetheless, cargo-bikes still belong to a niche-market, and few studies on them have been conducted.

In Norway, the prevalence of bike possession is approximately 75%, yet the annual cycling share is only 5%, with seasonal variations ranging from 1% in the winter to 8% in the summer [27]. Hence, it should be manageable to drive less and cycle more. In 2014, 59% of all trips between one and 2.9 kilometers (km) were conducted by car and 8% by bike, while the average distance of bike trips was 5.1 km [27]. However, for parents of young children, most factors influencing mode choice seem to facilitate car use, e.g. both parents being in paid work, organized leisure activities (for older siblings), increased perceived time pressure and enhanced car access [28]. Traveling to kindergarten by bike rather than by car could potentially teach children that alternative modes of transport exist, thereby encouraging future healthy and sustainable transportation and PA habits.

Understanding the essential determinants for the behavior or the activity of interest would increase the chances of developing effective intervention schemes and persistent behavior change. Travel has traditionally been considered externally motivated, e.g. as a means to move from one destination to another, but intrinsic motivation has gained increased attention [29]. According to the Self-determination Theory (SDT) [30], the degree to which the basic needs of autonomy, competence and relatedness are met would determine motivation on a continuum ranging from extrinsic motivation to intrinsic motivation [31]. Extrinsic motivation focuses on consequences that are separate from engaging in the specific activity, such as satisfying external pressures or achieving rewards, while intrinsic motivation is based on interest and engagement in the activity itself [31]. In order to sustain behaviors, intrinsic motivation should be strived for. Current knowledge supports the value of SDT in understanding and promoting exercise behavior [32], and higher levels of intrinsic motivation have been related to greater levels of active transport [33]. Moreover, the Theory of Planned Behavior (TPB) is commonly used to predict behaviors based on individuals’ intentions to perform the behavior of interest. In turn, these intensions are determined from attitudes towards the behavior, subjective norms, and perceived behavioral control [34]. Studies have linked active transport (cycling and walking) to these psychological constructs, i.e. attitudes, subjective norms, perceived behavioral control and intentions [35–37]. However, habit strength could moderate the impact of intentions on actual behavior, and has therefore been included in more extensive models assessing behaviors like travel mode choice [38]. Also, human behavior is commonly affected by environmental determinants, like accessibility [39]. Supporting this, access to bikes and accompanying equipment is suggested to be a key factor associated with transport cycling [40]. Correspondingly, a British study providing e-bike access for six to eight weeks was seen to influence employees’ travel behaviors and total PA level substantially, including in employees not usually engaged with PA, or feeling unable to use a conventional bike [41]. Bike share programs represents another aspect of accessibility, and a number of cities have now introduced e-bike sharing potentially encouraging new users to bike share [42].

To the best of our knowledge, no previous studies have tested the effects of providing free access to different bike types for a prolonged period with regard to the parents of young children. Therefore, the primary objective of the present study was to assess the feasibility and effect of an intervention providing participants with access to an e-bike including a trailer for child transportation, a cargo (longtail) bike, and a traditional bike including a trailer—each bike type for three months—on transportation habits (weekly frequency of cycling and driving to the workplace, the kindergarten and the grocery store) and total cycling (distance and time). The secondary objective was to assess possible intervention effects on intrinsic motivation for cycling, and psychological constructs and habit strength related to car use.

## Methods

### Study design

A randomized controlled trial was conducted in a real-life setting in Southern Norway [43], from September 2017 to June 2018 (S6 and S7 Tables). The project leader (EB) and the project coordinator (HBB) matched participants (*n* = 36) in pairs according to sex, cardiorespiratory fitness and height, prior to randomly drawing participants into an intervention group or a control group. Firstly, participants were split according to sex, to have nine males and nine females in each group. Secondly, both males and females were ranked according to cardiorespiratory fitness and height (from highest to lowest), and further matched in pairs. The rationale for matching participants according to cardiorespiratory fitness was to ensure as equal groups as possible. Matching according to height was for practical reasons, that is, we did not have the opportunity to provide each participant with a new bike customized for him/her. Participants in the intervention group (*n* = 18) completed the following intervention arms in random order: (i) three months’ access to an e-bike with trailer (*n* = 6), (ii) three months’ access to a longtail bike (*n* = 6), and (iii) three months’ access to a traditional bike with trailer (*n* = 6), in total nine months. The intervention arms followed the autumn (September-November), winter (December-February) and spring (March-May) seasons, respectively. No instructions as to the amount of cycling were given. Participants in the control group (*n* = 18) were requested to maintain usual transportation and PA habits. The present study was conducted according to the guidelines in the Declaration of Helsinki [44], and research clearance was assigned by the Norwegian Center for Research Data (NSD). All participants were given written information about study objectives and methods prior to providing consent electronically. The trial was registered at clinicaltrials.gov, with number NCT03131518, URL https://clinicaltrials.gov/ct2/show/NCT03131518.

Furthermore, a protocol paper was published [43].

### Bike types and trailers

The e-bikes were of the type Emotion Neo Cross/Neo Jet (BH Bikes, Vitoria, Spain), 2012-model with a Samsung 432 Wh battery, wheel size 28”, and a bike weight of 21.8 kg. The motor capacity was 350 W, and maximal speed was 25 km/h (with an active engine). The longtails were the model Surly Big Dummy (Surly Bikes, Minnesota, US), 2010–2017 models with SRAM GX/Shimano Deore/XT gear shifters (8 speed/10-speed), wheel size 26” and bike weight of 21.8 kg (including rails, deck and bags), and 26.6 kg when also including one child seat. The load capacity of the Surly Big Dummy is 400 lb (181 kg), including the rider. The traditional bikes were two different models; DBS Rallar Flåm (DBS, Taiwan), 2013 model with an internal Shimano Nexus 7 speed hub, wheel size 28” and bike weight of 13.5 kg, and one Kalkhoff Jubilee (Kalkhoff, Cloppenburg, Germany), 2017 model with an internal Shimano Nexus 7 speed hub, wheel size 28” and bike weight of 13.5 kg. Two participants requested and were permitted to use their own traditional bikes, entailing the following bike types; White Pro, 2013 model with Shimano Ultegra gear shifters (11-speed), wheel size 28” and bike weight of 9.5 kg, and Cube LTD Pro 29 (Cube bikes, Waldershof, Germany), 2013 model with Shimano SLX gear shifters (10-speed), wheel size 29” and bike weight of 12.6 kg. The bike trailers were of the type Spectra Eco (Cycleurope, Stockholm, Sweden), with a steel frame, wheel size 20”, a weight of 14 kg, and a load capacity of 30 kg (maximum total weight of 45 kg). Participants were offered technical assistance from a bike repair shop throughout the trial period. Bike helmets for both parent and child, a safety vest, and lights were provided, and during the winter season the bikes were equipped with winter tires with studs. Participants received and returned bikes and equipment at the University of Agder, Campus Kristiansand.

### Study sample

**Fig 1:**
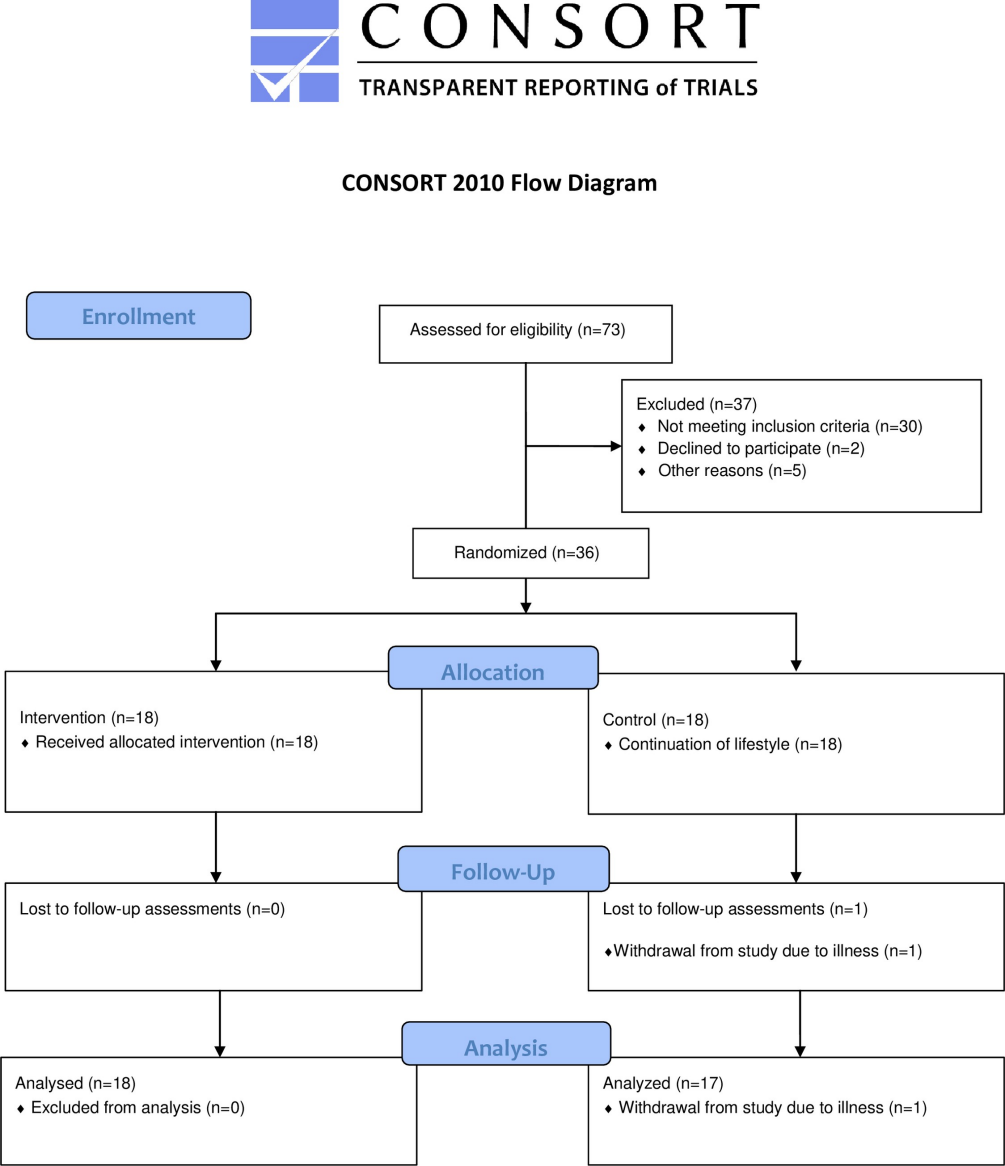
Participants flow. The study was conducted in Kristiansand, Norway, from September 2017 to June 2018.

A convenience sample was recruited, consisting of 36 parents (Fig 1) with children attending kindergarten and residing in Southern Norway. Recruitment was carried out via Facebook announcements and direct contact with kindergartens and selected organizations and businesses in Kristiansand municipality from May through August 2017. The main outcome was cycling amount (distance and time). Based on a standard deviation (SD) of 60 minutes (min) per week [45], a power of 0.80 and a significance level of 5%, a sample size of 16 subjects in the intervention group and 16 subjects in the control group would allow for detecting an increase in cycling time from 15 min to 75 min per week (i.e. half of weekly PA recommendations). This meant that we aimed to recruit participants that cycled less than 15 minutes per week initially. To account for a 10% drop-out, and to utilize the bikes optimally according to the study design, a total of 36 participants were enrolled by the project coordinator (HBB). Inclusion criteria were (i) being able to understand and read Norwegian, (ii) having one child born in year 2013, 2014 or 2015 attending kindergarten, (iii) being responsible for bringing to/picking up the child from kindergarten ≥5 times per week, or at least half of the time, (iv) residing 2–10 km from the workplace (i.e. excluding those working from home), (v) residing <3 km from the kindergarten and the grocery store, (vi) having car access, (vii) possessing a smartphone, (viii) being between 167–190 centimeters (cm) tall (due to the size of the accessible bikes), and (ix) having the opportunity to store the bikes indoors. Exclusion criteria were: (i) being physically active, i.e. meeting the PA recommendations, (ii) having cycled more than once weekly throughout the last twelve months to the workplace, kindergarten or grocery store, and (iii) suffering from severe cardiovascular diseases or upper respiratory tract diseases.

### Measurements

#### Cycling

Cycling distance and time were measured continuously with a bicycle computer (CatEye Velo 9, CatEye, Osaka, Japan), and recorded by each participant per day of cycling (i.e. a recording per trip was not required). In addition, cycling data was recorded by the project coordinator every third month, i.e. after each intervention arm.

#### Questionnaire data

Participants answered a web-based questionnaire when signing up and providing consent for study participation. The questionnaire assessed relevant background information such as sex, date of birth, ethnicity and educational level, together with information determining eligibility for inclusion (e.g. cycling frequency the past 12 months and habitual PA-level) (S2 Table). In addition, a questionnaire assessing transportation habits, intrinsic motivation, barriers and facilitators for cycling, as well as psychological constructs (attitudes, subjective norms, perceived behavioral control and intentions) and habit strength related to car use, was filled in by the intervention group at baseline and after each intervention arm, in total four times. The control group answered the questionnaire at baseline and at the nine-month follow-up, i.e. at intervention completion. Prior to data collection, both questionnaires were pilot-tested for comprehensibility in seven subjects representative for the target group, and questions were slightly adapted. Completing the questionnaires took 10–20 min. Data collection was performed between August 2017 and June 2018, in short periods following the intervention arms.

Transportation habits were at baseline measured as usual frequency of walking, cycling, driving a car and using public transport to the workplace (days/week; 0–5), the kindergarten (trips/week; 0–10) and the grocery store (days/week; 0–5), for the autumn, winter and spring season, respectively.[46]. For the kindergarten, bringing and picking up the child were assessed independently, as these are conducted as separate trips. Then participants were classified into main mode of transport (i.e. walkers, cyclists, car users or public transport users) if >50% of weekly days/trips were conducted by that mode [46]. For the follow-up questionnaires filled in after 3, 6 and 9 months, only travel behaviour for the preceding three months was assessed (i.e. travel behavior during each intervention arm). The control group answered the questionnaire at baseline and at nine-month follow-up only.

Intrinsic motivation for cycling was assessed with the Intrinsic Motivation Inventory, being a measurement tool commonly used to assess intrinsic motivation and self-regulation related to specific activities [47], and the Behavioral Regulation in Exercise Questionnaire 2 [48, 49]. For the Intrinsic Motivation Inventory, three out of seven subscales were selected; interest/enjoyment, value/usefulness and perceived choice (S3 and S4 Tables). The Behavioral regulation in exercise questionnaire 2 explores the continuum of behavioral regulation (from external to intrinsic motivation) [48], in addition to amotivation [49], to gain increased understanding of motivation for exercise. Psychological constructs related to car use, i.e. attitude, subjective norms, perceived behavioral control and intention [34] were assessed based on a previously validated questionnaire [50], and a more comprehensive action determination model including perceived mobility necessities as part of the perceived behavioral control measure [38]. Further, habit strength for car use was explored using seven statements from the Habit Strength Index [51, 52] (S5 Table).

### Data analyses

The statistical analyses were performed using the software package IBM SPSS Statistics version 24.0 (IBM Corp., Somers, New York, USA). A two-sided p-value of ≤0.05 was considered statistically significant. Descriptive analyses were conducted, and continuous variables are presented as means and standard deviations (SD), or median and interquartile range (IQR) for skewed data, while categorical variables are presented as numbers and proportions. The small sample size and skewed data distribution did not allow for regression analyses or parametric methods. Hence, non-parametric methods were used to assess differences between groups (intervention vs. control) at baseline and at the nine-month follow-up, and for change from baseline to the nine-month follow-up. Crosstabs (Fisher’s Exact test) were used for the dichotomous variable “mode classification”, and the Mann-Whitney U-test for the continuous variables concerning intrinsic motivation and psychological constructs related to car use. Changes within groups for frequencies of cycling and driving were tested with the Wilcoxon Signed Rank test.

## Results

Baseline characteristics across intervention and control group participants are presented in Table 1, showing that groups were not significantly different at baseline.

**Table 1:** Baseline characteristics across intervention and control group participants.

	Intervention group (*n* = 18)	Control group (*n* = 18)	*p-value
**Age; years (mean (SD))**	35.8 (5.0)	35.5 (4.0)	0.85
**^§^Ethnicity; native Norwegian (n (%))**	16 (89.0)	12 (67.0)	0.23
**^†^Educational level; high (n (%))**	10 (56.0)	9 (50.0)	0.99
**Body mass index; kg/m^2^ (median (IQR))**	24.7 (4.2)	24.2 (6.3)	0.68
**Distance to the workplace; km (median (IQR))**	7.1 (4.9)	7.1 (4.3)	0.85
**Distance to the kindergarten; km (median (IQR))**	1.3 (1.1)	1.5 (2.6)	0.99
**Distance to the grocery store; km (median (IQR))**	1.4 (1.1)	1.5 (1.3)	0.95

IQR = interquartile range

*P-values were calculated using Chi-square test for categorical data, Independent samples t-test for continuous data (age) and Mann-Whitney U-test for continuous but skewed data (body mass index and distance). A two-sided p-value of ≤0.05 was considered statistically significant.

^§^Participant and both parents born in Norway.

^†^≥4 years of college or university education.

### Travel behavior

**Fig 2:**
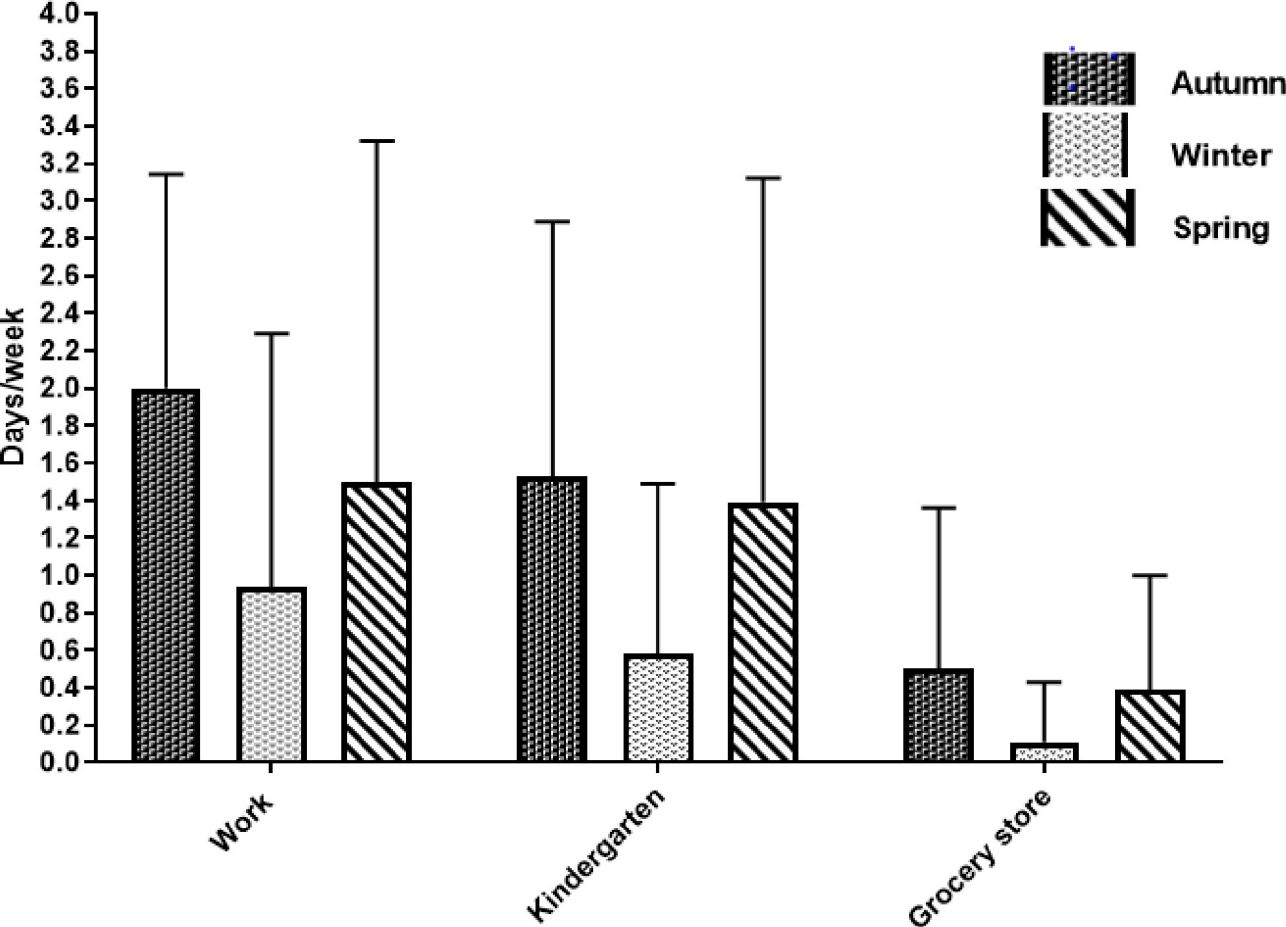
Change in cycling frequency. Change (mean (SD), days/week) in cycling frequency from baseline to follow-up in the intervention group (n = 18), grouped according to destinations (work, kindergarten, grocery store) for the autumn, winter and spring seasons respectively. Follow-up measures were conducted after 3, 6 and 9 months, i.e. following the autumn, winter and spring season.

**Fig 3:**
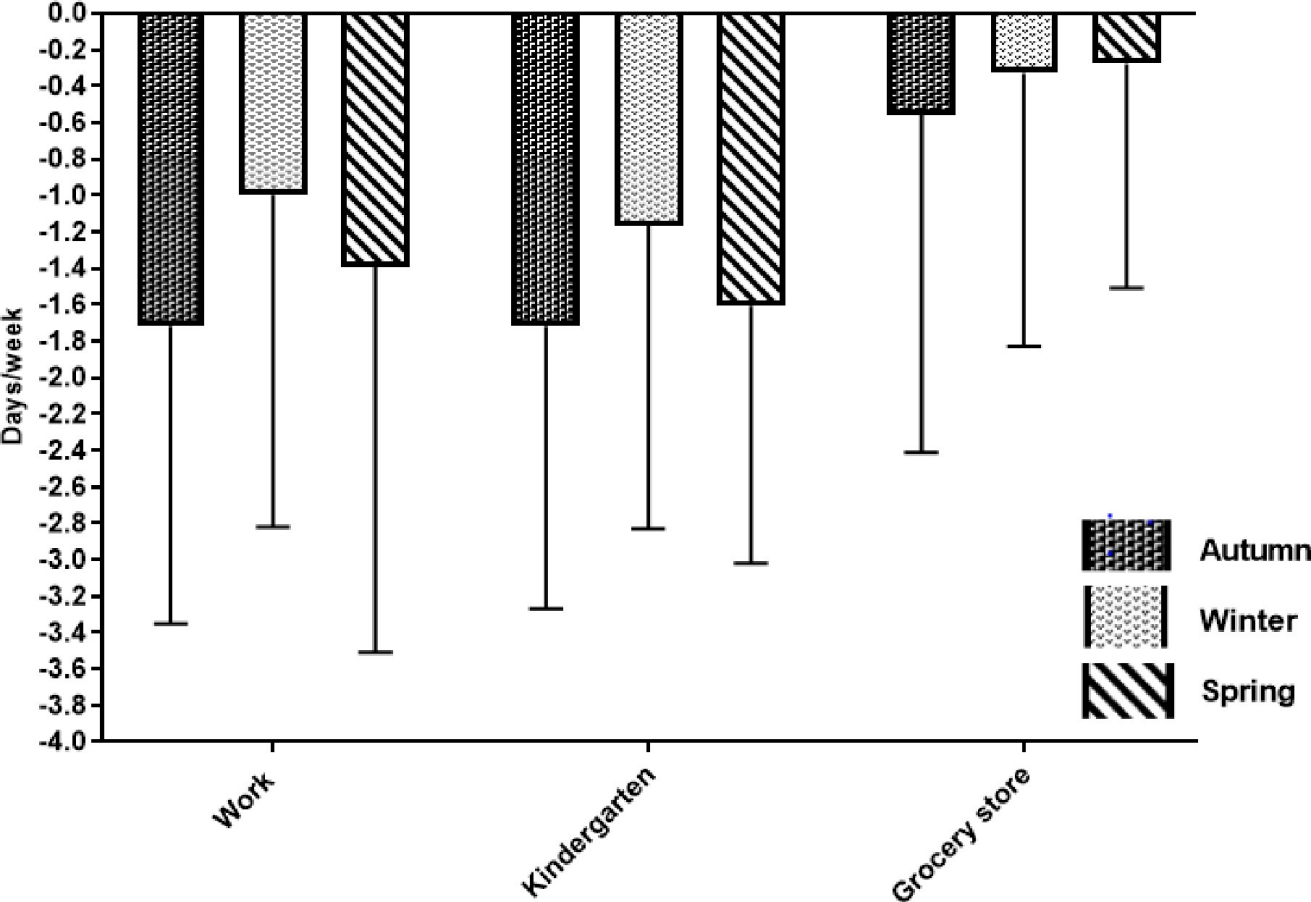
Change in frequency of driving. Change (mean (SD), days/week) in frequency of driving a car from baseline to follow-up in the intervention group (n = 18), grouped according to destinations (work, kindergarten, grocery store) for the autumn, winter and spring seasons respectively. Follow-up measures were conducted after 3, 6 and 9 months, i.e. following the autumn, winter and spring season.

At the nine-month follow-up significantly more participants from the intervention group (compared with the control group) were classified as cyclists to the workplace (*n* = 7 (38.9%) vs. *n* = 1 (5.9%), *p* = 0.04), yet not to the kindergarten (*n* = 6 (33.3%) vs. *n* = 2 (11.8%), *p* = 0.23) or to the grocery store (*n* = 2 (11.1%) vs. *n* = 0 (0%), *p* = 0.49). No differences in mode classification between groups were found for being a car user (Table 2). For the intervention group, a significant increase in cycling frequency from baseline to the nine-month follow-up was found for all destinations and seasons, except to the grocery store during the winter season (*p* = 0.16). Change in cycling frequency ranged from 0.1 (“grocery store, winter”) days per week to 2.0 (“work, autumn”) days per week (Fig 2). For frequency of driving a car, a significant decrease was observed for the travel to the workplace and the kindergarten for all seasons, yet not for the grocery store for any season (*p* = 0.15–0.49). Change in frequency of driving ranged from -0.2 (“grocery store, spring”) days per week to -1.7 (“work, autumn” and “kindergarten, autumn”) days per week (Fig 3). For the control group, no significant changes were observed for frequency of cycling or for driving.

**Table 2:** Mode classification across intervention and control group participants.

Total	Cyclist *n* (%) Baseline	*p-value	Car user *n* (%) Baseline	*p-value	Cyclist *n* (%) Nine-month follow-up	*p-value	Car user *n* (%) Nine-month follow-up	*p-value
**Workplace** Intervention (*n* = 18) Control (*n* = 18)								
**Intervention (*n* = 18)**	0 (0)	0.99	15 (83.3)	0.99	7 (38.9)	0.04	9 (50.0)	0.16
**Control (*n* = 18)**	1 (5.6)		16 (88.9)		1 (5.9)		13 (76.5)	
**Kindergarten**								
Intervention (*n* = 18)	0 (0)	0.49	17 (94.4)	0.34	6 (33.3)	0.23	11 (61.1)	0.26
Control (*n* = 18)	2 (11.1)		14 (77.8)		2 (11.8)		14 (82.4)	
**Grocery store**								
Intervention (*n* = 18)	0 (0)	NA	16 (88.9)	0.66	2 (11.1)	0.49	13 (72.2)	0.69
Control (*n* = 18)	0 (0)		14 (77.8)		0 (0)		14 (82.4)	

Participants were classified as cyclists or car users if >50% of weekly days/trips to the different destinations were conducted using that mode. Data concerns the spring season at both baseline and at nine-month follow-up.

*P-values were calculated using Crosstabs (Fisher’s Exact test). A two-sided p-value of ≤0.05 was considered statistically significant. At follow-up 17 participants from the control group completed the questionnaire.

### Cycling within the intervention group

There were major individual differences in cycling among the participants in the intervention group, with cycling distance for the total trial ranging from 44 km to 1408 km, with a weekly median of 10.9 km (IQR 20.8 km). In total 6 (33.3%) participants cycled ≥75min per week throughout the entire trial period (42.9 min (65.3 min), median (IQR)). Further, for the trial period in total, the traditional bikes accrued 2798 km and 11 503 min of cycling, the longtail bikes 2750 km and 10 572 min, and the e-bikes 4164 km and 12 371 min, entailing an average speed of 14.6, 15.6 and 20.2 km per hour respectively. On the individual level, cycling distance within each season ranged from 25 km to 561 km (traditional bike) for the autumn, from 0 km to 401 km (e-bike) for the winter, and from 3 km to 504 km (e-bike) for the spring. Further, cycling time per participant ranged from 26 mins to 1906 mins (traditional bike), from 0 min to 1351 mins (e-bike), and from 18 mins to 2015 mins (longtail) within the autumn, winter and spring seasons respectively. E-bikes achieved the greatest cycling amount (distance and time) for the entire trial period, with the smallest sample variability (IQR). During the first cycling period, i.e. the autumn season, the traditional bikes achieved the greatest amount of cycling per week (Table 3).

**Table 3:** Cycling distance and time per week for the total trial and for each season across bike types.

	Cycling distance per week (km) median (IQR)	Cycling time per week (min) median (IQR)
**Trial period in total**		
E-bike (*n* = 18)	20.2 (24.8)	62.7 (68.5)
Longtail (*n* = 18)	9.3 (21.1)	40.0 (72.7)
Traditional bike (*n* = 18)	11.9 (21.2)	51.1 (84.7)
**Autumn**		
E-bike (*n* = 6)	16.1 (16.6)	44.9 (40.6)
Longtail (*n* = 6)	18.6 (31.5)	83.0 (105.5)
Traditional bike (*n* = 6)	26.3 (37.4)	108.5 (132.3)
**Winter**		
E-bike (*n* = 6)	11.2 (25.2)	41.3 (74.9)
Longtail (*n* = 6)	3.2 (15.1)	14.6 (48.1)
Traditional bike (*n* = 6)	2.3 (5.3)	10.7 (24.8)
**Spring**		
E-bike (*n* = 6)	33.3 (32.6)	101.9 (89.9)
Longtail (*n* = 6)	6.2 (16.6)	22.5 (64.6)
Traditional bike (*n* = 6)	7.0 (21.0)	34.1 (97.1)

IQR = interquartile range

### Intrinsic motivation for cycling and psychological constructs related to car use

The intervention group reported significantly higher “intrinsic regulation” for cycling at the nine-month follow-up, compared with the control group (*p* = 0.01). No other differences were found across groups for the motivational aspects related to cycling, or for the psychological constructs related to car use (S1 Table). Regarding the change from baseline to the nine-month follow-up, scoring for “interest/enjoyment”, “introjected regulation” and “intrinsic regulation” for cycling increased in the intervention group, and remained stable or decreased in the control group (*p* = 0.02, *p* = 0.01 and *p* = 0.02, respectively) (S1 Table).

## Discussion

The present trial, providing participants with access to different bike types for in total nine months, resulted in increased cycling and decreased car use in parents with children attending kindergarten, as well as higher intrinsic motivation for cycling. These findings agree with the socio-ecological framework [39], claiming that accessibility is one relevant environmental determinant for PA, including cycling for transport [40]. Accordingly, yet targeting e-bikes only, a Norwegian real-life controlled experiment providing 66 participants with e-bikes for two or four weeks, generated considerably more cycling [19]. Fyhri and Fernley reported the number of cycling trips to increase from 0.9 to 1.4 per day, distance cycled to increase from 4.8 km to 10.3 km, and cycling shares out of all transport to increase from 28% to 48% [19]. Likewise, Cairns et al. [41] found that after loaning 80 employees an e-bike for six to eight weeks, car mileage was reduced by 20%, 59% of the employees increased their overall PA, whilst 73% would cycle to work at least once a week, if they had an e-bike accessible [41]. Concerning cargo bikes specifically, American data has shown a decrease in car travel among cargo bike owners [22]. Also, if conditions facilitate it, American mothers accessing a cargo bike seem to consider it a realistic option for trips usually made by car [26]. Regarding bike share, representing another means to bike access, a pilot study testing a university-based e-bike share in North-America reported that new users were attracted to cycling [53]. Multi-city analyses of regular bike shares’ impact on car use and PA suggest that car use decreases, yet this is of limited magnitude [54]. Nevertheless, PA levels could increase [55] due to mode shifts following bike share usage.

E-bikes achieved the greatest cycling amounts for the trial period in total in the present study, which corresponds with previous studies reporting e-bike users to cycle longer distances, and more often [18–21]. It is likely that increased cycling with an e-bike is explained by its tendency to reduce typical barriers for cycling such as long distances, hilly terrain, time use and the need to shower afterwards [19, 21, 56]. Also, e-bikes could be speed competitive with both public transport [57] and private cars [58]. Besides, seasonal variations that were previously reported to decrease commuter cycling with regular bikes significantly [59], may in fact become less problematic when assistance from an electric motor is provided [57]. The heavy weight of an e-bike, combined with the power, could offer more traction in winter conditions, hence it being easier to ride during all seasons [60]. Nevertheless, this would apply to a greater extent for experienced cyclists than for newcomers.

Even though cycling increased and car use decreased in the intervention group, there were major inter-subject differences. In Norway substantial seasonal variation is normal due to the climate, yet the winter season in the present trial was even more icy and snowy than usual in Southern Norway, making cycling more challenging. For subjects not used to cycling in particular, this likely affected cycling negatively, and may be one reason for the large individual differences. As suggested by the SDT [30], intrinsic motivation would increase the chances of behaviors being performed, and further that they would be sustained. In turn, increased compliance with the basic needs of autonomy, competence and relatedness would result in a move on the motivational continuum from more controlled regulations to autonomous regulation, with intrinsic motivation as the desired endpoint [31]. Following this rationale, it could be the case that those cycling even during the tough winter months experienced (unexpected) mastery, which in turn facilitated the basic need of competence, and further increased intrinsic motivation. Supporting this, the intervention group increased their intrinsic motivation for cycling throughout the trial period. However, we also found that scoring on “interest/enjoyment”, “introjected regulation” and “intrinsic regulation” for cycling increased in the intervention group and remained stable or decreased in the control group. The accompaniment of introjected regulation (being one controlled form of regulation) with intrinsic regulation may be somewhat surprising from a strict SDT perspective. Nevertheless, this finding agrees with previous work demonstrating that introjected regulation can follow intrinsic regulation, and further associate positively with exercise [61], total PA and active transportation [33]. It is noteworthy, though, that the amount of cycling was higher during the first period (autumn) than during the latter period (spring), with the greatest amount found for the traditional bikes. These findings may express fatigue and decreased motivation in some participants, as one might have expected the opposite trend in cycling based on season and weather conditions [62], as well as previous findings showing that e-bike users cycle more than those using a traditional bike [18].

For the present sample, switching from car to bike seemed the most challenging for the travel to the grocery store. Carrying capacity probably represents one key factor, together with combining more activities into one journey, as combined travels are found to be conducted by bike to a lesser extent than commuting trips separately [57]. The bike trailer complementing the traditional bike and the e-bike would allow for extra items to be carried if bringing one child, yet to a limited extent if bringing two children. The bags following the longtails could carry substantial loads, yet the feet protection on the child seat somewhat limited the carrying capacity of the bags. In addition, solid loads combined with child transportation entails considerable weight. Hence, for those not cycling regularly, nor being classified as physically active (due to inclusion criteria), the weight load may be too high. In turn, this could reduce the utility of the human-powered longtails.

### Strengths and limitations

One study strength was the natural setting of the intervention, i.e. access to bikes and equipment with no instructions on cycling amount. Thus, the effect of accessibility on voluntary cycling could be explored, and thereby the feasibility of cycling for transportation among parents with children in kindergarten. Another study strength was the use of a comprehensive and previously tested measurement tool [46] for assessing the traveling frequency of different modes of transportation to selected destinations across the annual seasons. On the other hand, the most substantial study limitation was that the sample size did not allow for regression analyses, and thus not for the adjustment of confounders. Power calculations were conducted, yet these calculations were based on change in cycling time for both groups (intervention and control) from baseline to follow-up measures. Initially, it was planned that both groups should be provided access to a smartphone application assessing transportation mode, trip purpose and cycling distance and time [43]. Nevertheless, this smartphone application could not be applied in the present trial due to delayed development, hence regular bicycle computers were used for recording cycling. Also, as the bicycle computers followed the bikes provided for the intervention group only, cycling data was recorded only for the intervention group. Furthermore, one participant experienced repeated technical issues with the e-bike during the second intervention arm (the winter season), affecting cycling negatively for this participant. In addition, two participants were permitted to use their own regular bike instead of the traditional bike provided in the project, resulting in diverse types of regular bikes being included. Concerning adverse events, injuries were not registered in the present trial. As this is recommended for exercise interventions, such a registration should have been conducted. Moreover, since a convenience sample was recruited, those who were highly educated were overrepresented, resulting in reduced generalizability to the general population of parents with children attending kindergarten.

### Perspectives

The present trial, entailing bike access as the only intervention component, contributes to enhanced insights into the feasibility of cycling for transportation for parents of young children in geographical and infrastructural contexts differing from those in typical cycling cultures like the Netherlands and Denmark. Also, the potential of different bike types for a broader scope than commuting alone, i.e. the travel to the kindergarten and the grocery store, is explored. One could claim that if it is possible in Norway, a country with low temperatures and snowy and icy winters, it should be highly possible in countries and regions with more cycling friendly climates, topographies and infrastructures.

Next to the topics addressed in the present study, the safety issue of cycling, especially for e-bikes, raises concerns. Therefore, topics such as e-bike involved crashes, e-bikes’ red light running behavior among others, should be examined in future studies.

## Conclusion

Access to different bike types for families with children in kindergarten resulted in overall increased cycling and decreased car use. Substantial individual differences in cycling likely express the importance of the motivational aspects, in addition to accessibility. For subjects who are not well-trained, e-bikes with trailers or electric assisted longtails may be more feasible and sustainable for transporting goods and children by bike than traditional bikes with trailers and human-powered longtails. Thus, providing parents with children in kindergarten with access to e-bikes might increase cycling for some, also during the winter season. Higher levels of intrinsic motivation for cycling may contribute to increased active travel, also in the long term.

## Supporting information

S1 Fig Cycling. Cycling distance across bike types, for each of the participants in the intervention group (n = 18), for each season. Figures are median and interquartile range.
(TIF)

S1 Table Intrinsic motivation. Intrinsic motivation for cycling and psychological constructs related to car use.
(DOCX)

S2 Table Baseline characteristics. Items assessing ethnicity, educational level, and self-reported cycling frequency and habitual physical activity level at inclusion.
(DOCX)

S3 Table The Intrinsic Motivation Inventory. Items included in selected Intrinsic Motivation Inventory (IMI)- subscales, assessing intrinsic motivation related to cycling.
(DOCX)

S4 Table The Behavioral regulation in Exercise Questionnaire 2. Items included in the subscales composing The Behavioral Regulation in Exercise Questionnaire (BREQ) 2, assessing type of motivation/motivational quality.
(DOCX)

S5 Table Psychological measures related to car use and habit strength for car use. (DOCX)

S6 Table CONSORT 2010 checklist. (DOC)

S7 Table Strobe TIDieR checklist. (DOCX)

S1 Dataset Data on background information, travel behavior and motivational aspects related to cycling and car use. (SAV)

S2 Dataset Data on cycling amount (time and distance) in the intervention group. (SAV)

S1 Text Project description, application for funding, project From cars to bikes. (DOCX)

## Abbreviations

MVPA: moderate-to-vigorous intensity physical activity
PA: physical activity
E-bike: Electric assisted bicycle
SDT: Self-determination theory
TPB: Theory of planned behavior
Km: kilometers
Min: minutes
Cm: centimeters
