# Supplementary figures and images for: From cars to bikes – The effect of an intervention providing access to different bike types: A randomized controlled trial
Source: PLoS One. 2019 Jul 10;14(7):e0219304. doi: 10.1371/journal.pone.0219304 (PMC6619759; doi:10.1371/journal.pone.0219304)

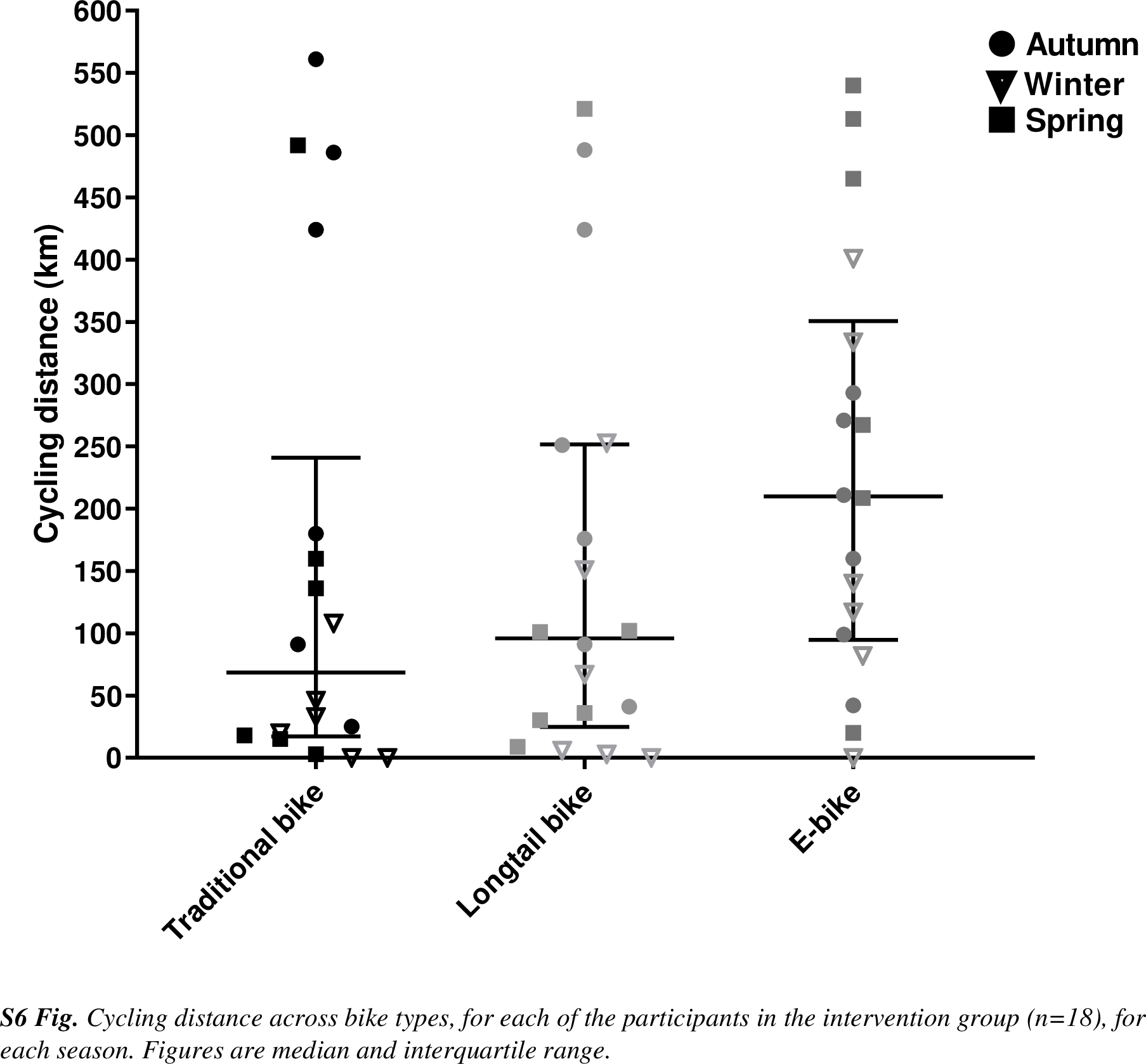

Supplement: S1 Fig — Cycling distance across bike types, for each of the participants in the intervention group (n = 18), for each season. Figures are median and interquartile range. (TIF) [file pone.0219304.s001.tif]
