# Supplementary material for: From cars to bikes – The effect of an intervention providing access to different bike types: A randomized controlled trial
Source: PLoS One. 2019 Jul 10;14(7):e0219304. doi: 10.1371/journal.pone.0219304 (PMC6619759; doi:10.1371/journal.pone.0219304)
Supplement: S1 Table — Intrinsic motivation for cycling and psychological constructs related to car use. (DOCX) [file pone.0219304.s002.docx]

|  | Intervention group (*n*=18)  Baseline | Control group (*n*=18)  Baseline | *p-value | | Intervention group (*n*=18)  Nine-months follow-up | Control group (*n*=17)  Nine-months follow-up | *p-value | Change  intervention group (*n*=18) | Change control group (*n*=17) | *p-value |
| --- | --- | --- | --- | --- | --- | --- | --- | --- | --- | --- |
| ^§^IMI; 1-7 points (med (IQR))  Interest/enjoyment  Perceived choice  Value/usefulness | 5.1 (1.3)  5.6 (1.4)  5.5 (1.3) | 4.8 (1.6)  5.5 (0.9)  5.6 (1.7) | | 0.98  0.73  0.95 | 5.6 (2.2)  5.0 (1.0)  5.9 (2.4) | 4.7 (3.0)  4.4 (2.2)  5.3 (1.7) | 0.14  0.27  0.41 | 0.4 (1.0)  -0.7 (1.5)  0.4 (1.2) | -0.6 (1.9)  -1.0 (1.5)  0.0 (1.7) | **0.02**  0.61  0.64 |
| ^†^BREQ; 0-4 points (med (IQR))  Amotivation  Extrinsic regulation  Introjected regulation  Identified regulation  Intrinsic regulation | 0.0 (0.75)  0.0 (0.0)  0.3 (0.7)  2.0 (1.5)  2.5 (1.5) | 0.0 (0.3)  0.3 (0.8)  0.7 (1.8)  2.1 (0.7)  2.4 (0.8) | | 0.41  **0.01**  **0.04**  0.52  0.44 | 0.0 (0.8)  0.0 (0.5)  1.0 (1.7)  2.4 (1.8)  3.4 (1.1) | 0.0 (1.0)  0.0 (0.6)  0.7 (1.0)  2.0 (1.9)  2.3 (1.7) | 0.40  0.40  0.35  0.31  **0.01** | 0.0 (0.1)  0.0 (0.1)  0.7 (1.7)  0.3 (1.4)  0.6 (0.8) | 0.0 (00.5)  0.0 (0.8)  0.0 (1.1)  0.0 (1.5)  0.0 (1.5) | 0.09  0.25  **0.01**  0.09  **0.02** |
| ^¶^Psychological constructs related to car use; 1-10 points (med (IQR))  Attitude  Perceived behavioral control  Social norms  Intention | 9.0 (2.0)  8.5 (1.5)  6.0 (5.0)  7.5 (3.0) | 9.0 (3.0)  9.0 (1.7)  6.0 (3.0)  7.5 (3.3) | | 0.72  0.86  0.70  0.91 | 3.5 (3.8)  4.0 (4.1)  6.0 (5.6)  8.0 (7.0) | 4.0 (1.0)  4.0 (1.5)  6.0 (3.0)  8.0 (2.0) | 0.92  0.71  0.88  0.55 | -5.0 (3.0)  -4.5 (1.8)  -1.0 (3.0)  0.0 (5.3) | -5.0 (4.0)  -4.5 (3.0)  -1.0 (2.5)  0.0 (3.0) | 0.83  0.79  0.46  0.59 |
| Habit strength for car use; 1-5 points  (med (IQR)) | 4.3 (0.8) | 4.4 (0.9) | | 0.66 | 3.9 (3.7) | 4.0 (1.5) | 0.46 | -0.5 (1.2) | 0.0 (1.1) | 0.20 |

**S1 Table** Intrinsic motivation for cycling and psychological constructs related to car use across intervention and control group participants, at baseline and at nine-month follow-up.

IQR=interquartile range

*P-values were calculated using Mann-Whitney U-test (skewed data). A two-sided p-value of ≤0.05 was considered statistically significant.

^§^Scale scores are based on the Intrinsic Motivation Inventory (IMI), and the selected subscales Interest/enjoyment, Perceived choice and Value/usefulness (1-7 points).

^†^Scale scores are based on Self Determination Theory and the Behavioral Regulation in Exercise Questionnaire 2 (BREQ 2).

^¶^Psychological constructs (attitude, perceived behavioral control, social norms, intention) are derived from the Theory of planned behavior (TPB), range 1-10 points (two summarized items, each 1-5 points). Higher scoring favors car use, i.e. not positive here. Habit strength for car use is based on the Habit strength index, with higher scoring expressing greater habit strength for car use (not positive).
