# Supplementary material for: From cars to bikes – The effect of an intervention providing access to different bike types: A randomized controlled trial
Source: PLoS One. 2019 Jul 10;14(7):e0219304. doi: 10.1371/journal.pone.0219304 (PMC6619759; doi:10.1371/journal.pone.0219304)
Supplement: S2 Table — Items assessing ethnicity, educational level, and self-reported cycling frequency and habitual physical activity level at inclusion. (DOCX) [file pone.0219304.s003.docx]

**S2 Table.** Items assessing ethnicity, educational level and self-reported cycling frequency and habitual PA level at inclusion.

| Construct being assessed | Items | Response alternatives and coding | Categorization |
| --- | --- | --- | --- |
| Ethnicity | Were you born in Norway?  Were both of your parents born in Norway? | no=0  yes=1  I don’t know=0  no, none of them=0  no, only one=0  yes, both=1  I don’t know=0 | 0=non-native  1=native Norwegian |
| Educational level | What is your highest completed education? | less than primary school (9-10 years)=0  primary school (9-10 years)=0  secondary school=0  college/university, <4 years=0  college/university, ≥4 years=1  others, please specify=0 | 0=low educational level  1=high educational level |
| Cycling frequency | The last 12 months, have you cycled more than once weekly to  -the workplace?  -the kindergarten?  -the grocery store? | no, I have cycled less=0  yes=1 | 0=non-cyclist (eligible for inclusion)  1=cyclist (not eligible for inclusion) |
| Habitual level of physical activity | How many days per week are you physically active for at least 30 minutes, in bouts of at least 10 minutes? * | none=0  one=0  two=0  three=0  four=0  five=1  six=1  every day=1 | 0=not physically active (eligible for inclusion)  1=physically active (not eligible for inclusion) |

*Physical activity was explained as “..all activity that makes your heart beat faster than usual, and you become short of breath occasionally, e.g. brisk walking, ballgames, or more targeted exercise like running, strength training, aerobics/dancing.”
