# Supplementary material for: From cars to bikes – The effect of an intervention providing access to different bike types: A randomized controlled trial
Source: PLoS One. 2019 Jul 10;14(7):e0219304. doi: 10.1371/journal.pone.0219304 (PMC6619759; doi:10.1371/journal.pone.0219304)
Supplement: S3 Table — Items included in selected Intrinsic Motivation Inventory (IMI)- subscales, assessing intrinsic motivation related to cycling. (DOCX) [file pone.0219304.s004.docx]

**S3 Table** Items included in selected Intrinsic Motivation Inventory (IMI)- subscales, assessing intrinsic motivation related to cycling for transportation.

| Subscale | Included items | Response alternatives and coding | Scoring |
| --- | --- | --- | --- |
| Interest/enjoyment | For each of the following statements concerning cycling for transportation, please indicate how true it is for you:  - I enjoyed cycling for transportation very much.  - Cycling for transportation was fun to do.  - I thought cycling for transportation was a boring activity. (R)  - Cycling for transportation did not hold my attention at all. (R)  - I would describe cycling for transportation as very interesting.  - I thought cycling for transportation was quite enjoyable.  - While I was cycling for transportation, I was thinking about how much I enjoyed it. | 1=not at all true  2  3  4=somewhat true  5  6  7=very true | 1: Negatively worded items (R) were scored reversed, i.e. the item response was subtracted from 8, with the resulting number representing the item score.  2: Subscale scores were calculated by averaging across all items on that subscale.  Possible scoring range: 1-7 points. |
| Perceived choice | - I believe I had some choice about cycling for transportation.  - I felt like it was not my own choice to cycle for transportation. (R)  - I didn’t really have a choice about cycling for transportation. (R)  - I felt like I had to cycle for transportation. (R)  - I cycled for transportation because I had no choice. (R)  - I cycled for transportation because I wanted to.  - I cycled for transportation because I had to. (R) |  |  |
| Value/usefulness | - I believe cycling for transportation could be of some value for me.  - I think cycling for transportation is useful.  - I think cycling for transportation is important to do.  - I would be willing to cycle more for transportation because it has some value to me.  - I think cycling for transportation could be helpful.  - I believe cycling for transportation could be beneficial to me.  - I think cycling for transportation is an important activity. |  |  |
