# Supplementary material for: From cars to bikes – The effect of an intervention providing access to different bike types: A randomized controlled trial
Source: PLoS One. 2019 Jul 10;14(7):e0219304. doi: 10.1371/journal.pone.0219304 (PMC6619759; doi:10.1371/journal.pone.0219304)
Supplement: S4 Table — Items included in the subscales composing The Behavioral Regulation in Exercise Questionnaire (BREQ) 2, assessing type of motivation/motivational quality. (DOCX) [file pone.0219304.s005.docx]

**S4 Table** Items included in the subscales composing The Behavioral Regulation in Exercise Questionnaire (BREQ) 2, assessing type of motivation/motivational quality related to cycling for transportation.

| Subscale | Included items | Response alternatives and coding | Scoring |
| --- | --- | --- | --- |
| Amotivation | For each of the following statements concerning cycling for transportation, please indicate how true it is for you:  - I don’t see why I should have to cycle for transportation.  - I can’t see why I should bother cycling for transportation.  - I don’t see the point in cycling for transportation.  - I think that cycling for transportation is a waste of time. | 0=not true for me  1  2  3  4=very true to me | Subscale scores were calculated by averaging across all items on that subscale.  Possible scoring range: 0-4 points. |
| External regulation | - I cycle for transportation because other people say I should.  - I take part in exercise because my friends/family/spouse say I should.  - I exercise because others will not be pleased with me if I don't.  - I feel under pressure from my friends/family to exercise. |  |  |
| Introjected regulation | - I feel guilty when I don't cycle for transportation.  - I feel ashamed when I do not cycle for transportation.  - I feel like a failure when I haven't cycled for transportation. |  |  |
| Identified regulation | - I value the benefits of cycling for transportation.  - It's important to me to cycle for transportation.  - I think it is important to make the effort to cycle for transportation.  - I get restless if I don't cycle for transportation. |  |  |
| Intrinsic regulation | - I cycle for transportation because it's fun.  - I enjoy cycling for transportation.  - I find cycling for transportation a pleasurable activity.  - I get pleasure and satisfaction from cycling for transportation. |  |  |
