# Supplementary material for: From cars to bikes – The effect of an intervention providing access to different bike types: A randomized controlled trial
Source: PLoS One. 2019 Jul 10;14(7):e0219304. doi: 10.1371/journal.pone.0219304 (PMC6619759; doi:10.1371/journal.pone.0219304)
Supplement: S5 Table — (DOCX) [file pone.0219304.s006.docx]

**S5 Table.** Items assessing psychological measures related to car use.

| Construct being assessed | Included items | Response alternatives and coding | Scoring |
| --- | --- | --- | --- |
| Attitude (TPB) | For each of the following statements concerning car use, please indicate to what extent you agree:  - Overall, it would be good to use a car  - It would be pleasant to use a car. | 1=strongly disagree  2=disagree  3=neither agree nor disagree  4=agree  5=strongly agree | Scoring was calculated by summing the items related to each construct.  Possible scoring range: 1-10 points. |
| Subjective norm (TPB) | - Most people who are important to me would support me using a car.  - Most people who are important to me think I should use a car. |  |  |
| Perceived behavioral control (TPB) | - It would be easy for me to use a car.  - I would be able to use a car.  *- Circumstances force me to use the car on my frequent trips.  - It would be difficult to manage my frequent trips with environmentally friendly means of transportation. |  |  |
| Intention  (TPB) | - I intend to use a car.  - I am likely to use a car. |  |  |
| Habit strength  (Habit strength index) | Please indicate to what extent you agree in the following statement:  Using a car in everyday life is something….  - I do frequently.  - I do automatically.  - that would require effort not to do.  - that belongs to my daily routine.  - I would find hard not to do.  - that’s typically me.  - I have been doing for a long time. | 1=strongly disagree  2=disagree  3=neither agree nor disagree  4=agree  5=strongly agree | Scoring was calculated by averaging across all items.  Possible scoring range: 1-5 points |

TPB = Theory of planned behavior.

*Perceived mobility necessities were incorporated into the perceived behavioral control (PBC)-measure, according to Klöckner & Blöbaum (2010). Due to four included items, scoring of the PBC-measure was divided by 2 for standardization with the other measures, hence possible scoring ranged from 1-10 points for all TPB-constructs.
