# Supplementary material for: From cars to bikes – The effect of an intervention providing access to different bike types: A randomized controlled trial
Source: PLoS One. 2019 Jul 10;14(7):e0219304. doi: 10.1371/journal.pone.0219304 (PMC6619759; doi:10.1371/journal.pone.0219304)
Supplement: S7 Table — (DOCX) [file pone.0219304.s008.docx]

**The TIDieR (Template for Intervention Description and Replication) Checklist*:**


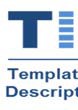

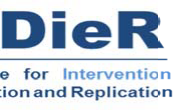


Information to include when describing an intervention and the location of the information

m

| **Item Item Where located **** | |
| --- | --- |
| **number** Primary paper  (page or appendix number) | Other ^†^ (details) |
| **BRIEF NAME**   1. Provide the name or a phrase that describes the intervention. p 1   **WHY**   1. Describe any rationale, theory, or goal of the elements essential to the intervention. p 4-6   **WHAT**   1. Materials: Describe any physical or informational materials used in the intervention, including those N/A provided to participants or used in intervention delivery or in training of intervention providers.   Provide information on where the materials can be accessed (e.g. online appendix, URL).   1. Procedures: Describe each of the procedures, activities, and/or processes used in the intervention, p 6-7 including any enabling or support activities.   **WHO PROVIDED**   1. For each category of intervention provider (e.g. psychologist, nursing assistant), describe their N/A expertise, background and any specific training given.   **HOW**   1. Describe the modes of delivery (e.g. face-to-face or by some other mechanism, such as internet or p 6-7 telephone) of the intervention and whether it was provided individually or in a group.   **WHERE**   1. Describe the type(s) of location(s) where the intervention occurred, including any necessary p 6-7 infrastructure or relevant features. |  |

|  | **WHEN and HOW MUCH** |  |
| --- | --- | --- |
| **8.** | Describe the number of times the intervention was delivered and over what period of time including | p 6-7 |
|  | the number of sessions, their schedule, and their duration, intensity or dose. |  |
|  | **TAILORING** |  |
| **9.** | If the intervention was planned to be personalised, titrated or adapted, then describe what, why, | N/A |
|  | when, and how. |  |
| **10.^ǂ^** | **MODIFICATIONS**  If the intervention was modified during the course of the study, describe the changes (what, why, | N/A |
|  | when, and how). |  |
|  | **HOW WELL** |  |
| **11.** | Planned: If intervention adherence or fidelity was assessed, describe how and by whom, and if any | N/A |
| **12.^ǂ^** | strategies were used to maintain or improve fidelity, describe them.  Actual: If intervention adherence or fidelity was assessed, describe the extent to which the | N/A |
|  | intervention was delivered as planned. |  |

** **Authors** - use N/A if an item is not applicable for the intervention being described. **Reviewers** – use ‘?’ if information about the element is not reported/not sufficiently reported.

† If the information is not provided in the primary paper, give details of where this information is available. This may include locations such as a published protocol or other published papers (provide citation details) or a website (provide the URL).

ǂ If completing the TIDieR checklist for a protocol, these items are not relevant to the protocol and cannot be described until the study is complete.

- We strongly recommend using this checklist in conjunction with the TIDieR guide (see *BMJ* 2014;348:g1687) which contains an explanation and elaboration for each item.
- The focus of TIDieR is on reporting details of the intervention elements (and where relevant, comparison elements) of a study. Other elements and methodological features of studies are covered by other reporting statements and checklists and have not been duplicated as part of the TIDieR checklist. When a **randomised trial** is being reported, the TIDieR checklist should be used in conjunction with the CONSORT statement (see www.consort‐statement.org) as an extension of **Item 5 of the CONSORT 2010 Statement.**

When a **clinical trial protocol** is being reported, the TIDieR checklist should be used in conjunction with the SPIRIT statement as an extension of **Item 11 of the SPIRIT 2013 Statement** (see www.spirit‐statement.org). For alternate study designs, TIDieR can be used in conjunction with the appropriate checklist for that study design (see www.equator‐network.org).
