# Supplementary material for: From cars to bikes – The effect of an intervention providing access to different bike types: A randomized controlled trial
Source: PLoS One. 2019 Jul 10;14(7):e0219304. doi: 10.1371/journal.pone.0219304 (PMC6619759; doi:10.1371/journal.pone.0219304)
Supplement: S1 Text — (DOCX) [file pone.0219304.s011.docx]

**From cars to bikes – the feasibility and effect of using e-bikes, traditional bikes and longtail bikes for transportation among parents of children attending kindergarten**

The current study aims to promote physical activity (PA) and health in parents of toddlers by giving access to e-bikes, longtail bikes and traditional bikes, to encourage replacement of non-active, motorized forms of transportation with active environmental friendly forms of transportation. There is a need for greater understanding of e-bikes and their role in the transportation network, and further effects on PA levels and health. Moreover, longtail bikes could meet certain practical needs not sufficiently fulfilled by e-bikes or traditional bikes, hence increased knowledge regarding their potential and feasibility should be obtained. No intervention study has investigated whether providing an e-bike or a longtail bike over an extended period in a sample of inactive parents of toddlers influence objectively assessed amount of cycling and total PA level, potential mode shifts, and effect on selected health parameters.

***Background***

*Physical activity levels and active transportation*

It is well documented that regular physical activity (PA) implies reduced risk for overweight and chronic diseases (1). Still, one third of adults and four fifths of adolescents globally do not reach PA guidelines (2), and recent Norwegian data showed that only 32 % of the adult population comply with the PA recommendations (3). Nonetheless, only 10 additional minutes of PA daily would make 2/3 of inactive persons adhere to current PA guidelines (4). For our ancestors, food procurement was inextricably linked to PA and energy expenditure (5), as they needed to hunt and forage in order to get food. In today’s society this link is broken- we can drive our car to the grocery store and buy the foods we need, with minor energy expenditure. In turn, these changes imply that being physically active today requires conscious choices to a larger degree. Physical inactivity is estimated to cause approximately 6-10% of the non-communicable diseases of coronary heart disease, type II diabetes, breast- and colon cancer, and 9 % of premature deaths worldwide, i.e. similar health effects as the established risk factors of obesity and smoking (6). Low cardiorespiratory fitness in itself, as a result of insufficient levels of PA, has; however, been reported to imply even greater mortality risk than obesity, diabetes type II and hypertension combined (7). Car use and other forms of motorized transportation favour neither health nor environmental sustainability as it entails sedentariness and emissions of greenhouse gases (GHGs). Active transportation i.e. walking or cycling for transportation purposes, may be a feasible and time efficient way to incorporate PA into daily routines, potentially increasing PA levels (8, 9). In turn, enhanced PA in forms of active transportation could promote health (8, 10-16), prevent obesity (17, 18), and decrease future healthcare costs (19). It has been calculated that increased PA would translate into significant health and socioeconomic gains (20). For bicycling specifically, a tripling in cycling in five urban areas in Norway between 2006-2009 has been estimated to entail health benefits of 250 million NOK annually (21). Next to direct effects on fitness and health, an additional advantage of active transportation is its potential to decrease GHG emissions (22, 23), as well as noise and pollution (8). Currently, about 23% of global climate gas discharges comes from motorized transportation (8). The ambitious goal of the Paris Agreement adopted by 195 countries in December 2015, entailing carbon neutrality before the end of the century (24), demands initiatives to be generated within all areas of society, not the least within the transportation sector. It is likely that an increased share of travels in Norway could be conducted as active transportation, considering that 25 % of daily travels done by car are shorter than 2.5 kilometers (21), and average distance of bicycle trips is 4 kilometers (25).

*Parents as facilitators of physical activity*

Lifestyle behaviors in childhood constitute the foundation for health throughout the lifespan, and research suggests that lifestyle habits, such as PA, track from childhood into adulthood (26, 27). Likewise, overweight and obese children are more likely to become overweight and obese adults, than normal weight children (28, 29). Parents are important facilitators and role models of PA in their kids, and among the significant correlates are parental PA, as well as parent participation in child PA (30). Being transported to kindergarten by bicycle instead of by car could teach children that alternative modes of transport exist, hence representing early adaptation to healthy and sustainable transportation and PA habits. Since parental PA behaviors are crucial for their own and their children’s current and future health, parents of toddlers is a target group of outmost importance. In terms of parental own PA habits, lack of time (31) and stress (32) are repeatedly documented to be negatively associated with PA in adults. In this regard, active transportation could potentially decrease the impact of time scarcity as a barrier, and may also reduce overall perceived stress through incorporating PA into daily transportation, i.e. less need for additional time consuming exercise.

*E-bikes and longtails*

Electric assisted bicycles (e-bikes) might represent an unexploited potential in terms of increased bicycle use. If replacing other motorized modes, and not replacing other PAs, e-bikes could favor both public health and the environment through increased levels of PA and decreased emissions of climate gases (23, 33). Sales of e-bikes in Europe increased nearly tenfold between 2007 and 2012 (34), while in Norway there was a twofold increase in sales numbers from 2014 to 2015, from 10 000 to about 20 000 bicycles (35). In 2015 e-bikes accounted for approximately 2% of total bicycle sales in Norway; a share which is expected to increase rapidly within the next years (35). In e-bikes categorized as pedelecs (power is activated by pedalling), propulsion is caused partly from the pedal- power of the rider, and partly from an electric engine providing power up to 25 km/h, or a maximum power output of 250 watt (36). It has been claimed that energy efficiency of an e-bike is greater than that of any other mode of transport, except from traditional bicycles (37). Besides, compared with regular bicycles, e-bikes enable maintenance of speed with less effort, which in turn helps overcoming some of the most common barriers to traditional pedal cycling, i.e. lack of fitness needed to cycle, hilly terrain, longer distances, lack of time, and lack of end of trip facilities (e.g. change rooms and shower) (34). A frequently reported motivation for purchasing an e-bike is the potential to replace motor vehicles, and findings across cities with high-quality transit systems tend to support this motivation (34). For illustration, a Chinese study based on four similar surveys in e-bike users over a 6-year period, reported that 24% and 55% of car and bus trips, respectively, were substituted with e-bike trips, while only 7% of trips from conventional bicycles were replaced (38). Thus, in this urban setting e-bikes seemed to function as an intermediate mode, interrupting the transition from bicycle to bus and from bus to car (38). Accordingly, in a sample of older Australians, the most frequent mode shift was from private cars to electric bicycles across all trip purposes (36). Moreover, current knowledge suggests that e-bike users cycle more often, and to more distant locations than those using regular bicycles (34, 37, 39), hence possessing greater capacity for exchanging car use than regular bicycles.

A major limitation with traditional bicycles, and also standard e-bikes, is the carrying capacity (40). At present there are several different cargo bikes on the market, both man-powered and with electric assistance, as well as various bike trailers for carrying goods and/or children. However, carrying stuff on a trailer may be less convenient than directly on a bike. In this regard, so-called longtail bikes (see picture) possess a great potential.


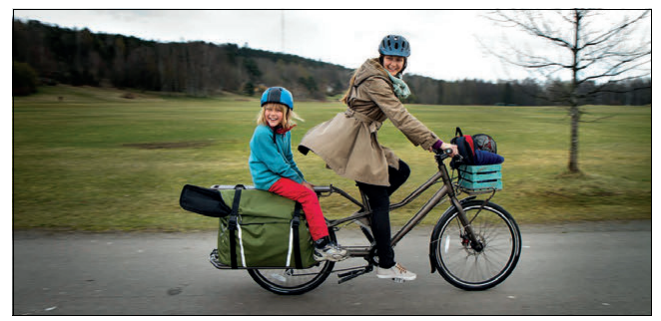


Figure1: A longtail bike in action. Source: Nasjonal Transportplan 2014-2023

Longtails are constructed to carry an adult, two children and additional luggage easily. Hence, longtail bicycles could potentially contribute to health promotion through increased cycling, and also a reduction in anthropogenic CO_2_ emissions related to motorized transportation, while simultaneously meeting a practical need not sufficiently accomplished by a traditional bike or e-bike. Such longtail bikes may be regarded as a re-linking of food procurement, and also child transportation, to energy expenditure. Considering that a great share of all travels are done within a limited range (21), longtail bikes might represent a feasible mode of transportation, yet there is currently a lack of scientific evidence. Our research group has tested longtail bikes in five families for a period lasting from five months up to five years. Out of these five families, two families registered their bicycle use in details for one year through filling in a diary. Hence, we acknowledge the great potential of such bikes for different purposes and for all seasons and weather conditions.

*Health effects of e-bicycling and use of longtails/transport bikes*

It is repeatedly found that both active (41) and inactive (42, 43) subjects reach moderate intensity PA levels when e-biking, i.e. levels sufficient for health benefits. Although e-bikes seem to entail lower intensity than traditional bikes, e-bikes could still boost overall levels of PA (34) and thereby promote health, if combined with more frequent and longer trips as proposed (37, 39). Nevertheless, current evidence regarding health effects of commuting with an e-bike is scarce, due to the lack of intervention studies assessing physiological parameters. De Geus and colleagues (44) conducted a quasi-experimental study in twenty untrained men and women, who were provided with an e-bike for six weeks. No change in maximum oxygen uptake (VO_2_ max) was found, yet a significant gain in maximal power output was achieved after six weeks of e-biking (44). Moreover, a pilot study conducted at the University of Agder (UiA) equipped 25 inactive adults with an e-bike for eight months, measuring participants′ VO_2_ max directly at baseline and at intervention determination. Results revealed an average 7.7% improvement in VO_2_ max, and cycling distance was positively associated with the increase, yet no control group was included (unpublished results). Also, focus group interviews were conducted and analyzed, revealing that e-biking contributed to very positive experiences regarding active commuting (unpublished results). In terms of potential health effects of using longtail or other transport bikes, no previous studies have addressed these associations. We assume though, that the intensity will be higher than for traditional bikes, entailing additional health effects if used as frequently as traditional bikes.

*Accessibility, social support and intrinsic motivation*

According to the ecological model provided by Sallis et al. (45) one important environmental determinant for PA, including active transportation, is accessibility. In line with this, a recent British study indicated that when made available, e-bikes could facilitate active travel and have substantial effects on travel behavior, also in subjects traditionally undertaking less PA or feeling unable to use a conventional bike (46). For the 80 subjects who borrowed bikes from their employer, 75% chose to use them at least once a week during their 6-8 week trial period. Across all participants, car mileage was reduced by 20%, and 59% reported that their overall PA increased. At the end of the trial, 73% said they would cycle to work at least once a week if they had an e-bike available (46). Bike share programs may be considered another aspect of accessibility, and more recently a number of cities have introduced e-bike shares, potentially encouraging new users to bike share (47). Underpinning this, a pilot study trialing a university based e-bike share in North-America reported that new users were attracted to cycling (48). Multi-city analyses of regular bike shares’ impact on car use and PA suggest that car use decreases, yet of limited magnitude (49). Nonetheless, PA levels could increase (50) due to mode shifts, further resulting in overall positive health effects (51). Like for accessibility, social support and the workplace environment could facilitate active transportation (45). A previous study by Wen and colleagues (52) assessing the role of workplaces in promoting active commuting, reported a significant inverse association between employees’ perception of workplace encouragement for active travel and driving to work. Also, physical support at work such as available bike parking and presence of showers, and cultural and social support for active transportation, has shown relevant for female employees’ transport choices (53). Accordingly, Yang et al. (54) found that worksite support and policies tended to associate with active commuting and the use of public transit. Together with previously reported higher income and education among e-bike owners (36), which may indicate that e-bikes are not affordable for all, current evidence and theory advocate that initiatives providing e-bike accessibility might increase e-bike usage. We hypothesize that increased accessibility and social support could facilitate intrinsic motivation for biking (55), through meeting the basic psychological needs of autonomy, competence and relatedness (56), and further result in higher levels of bicycling.

***Objectives***

We aim to assess the effect of an intervention where participants have access to an e-bike (including a trailer for transporting children, groceries and additional luggage), a longtail bike and a traditional bicycle (including a trailer), each bicycle for 4 months (in total 12 months), on the following aspects:

(1) Objectively assessed amount of biking, total levels of physical activity, and mode shifts from car/motorized modes to bicycle.

(2) Cardiorespiratory fitness, blood pressure, body composition, self-reported health and health-related quality of life (HRQoL).

(3) Experiences with bicycling (el/longtail/traditional) and intrinsic motivation for bicycling.

(4) How season and weather conditions influence the amount of bicycling, including potential differences across bicycle types.

(5) Potential spill-over effects on the participants’ partners.

***Methods***

*Design*

The present intervention study will have a cross-over design, entailing that all participants in the intervention group (*n*=18) will complete the following intervention arms in random order: (i) 4 months access to an e-bicycle with trailer (*n*=6), (ii) 4 months access to a longtail bicycle (*n*=6), and (iii) 4 months access to a regular bicycle with trailer (*n*=6), in total 12 months. Also, a control group (*n*=18) maintaining usual transportation and PA habits will be included. Randomization of participants into intervention or control group will be stratified according to sex and PA level. As incentive for participants randomized into the control group, those who fulfill the study will be in the draw of three traditional bicycles, including a trailer. Prior to study start written, informed consent will be obtained from all participants. To reduce the risk of accidents a bike helmet, a reflex vest, lights and winter tyres with spikes for the winter season will be handed out. The present study will apply The Norwegian Social Science Data Services (NSD) for ethical approval, and all participants will provide informed consent.

Table 1: Possible combination of intervention arms

| Month 1-4 | Month 5-8 | Month 9-12 |  |
| --- | --- | --- | --- |
| E-bike | Longtail | Traditional | *n* = 3 |
| E-bike | Traditional | Longtail | *n* = 3 |
| Longtail | E-bike | Traditional | *n* = 3 |
| Longtail | Traditional | E-bike | *n* = 3 |
| Traditional bike | E-bike | Longtail | *n* = 3 |
| Traditional bike | Longtail | E-bike | *n* = 3 |

*Study sample*

A convenience sample consisting of 36 inactive parents of toddlers will be recruited among employees at the University of Agder (UiA), campus Kristiansand. All measurements will be conducted in both the participant and the participant’s partner, in order to assess potential spill-over effects. Main outcome is total amount of bicycling (time and distance). Because of few previous studies in e-bikes and none in longtails targeting total amount of bicycling, power calculations are challenging to perform. However, based on a SD of 60 min/week (unpublished results at UiA), a power of 0.80 and a significance level of 5%, we will be able to detect an increase in cycling time from 15 min (based on the inclusion criteria) to 75 min a week (i.e. half of weekly PA recommendations) with 16 subjects in the intervention group and 16 subjects in the control group, which is clearly feasible. Yet, to account for 10% drop-out, and to utilize the bikes optimally per the study design, we will include 18 subjects in both groups (intervention and control). Inclusion criteria are: (i) one child born in 2014 attending kindergarten, (ii) being responsible for bringing/picking up the “study child” in the kindergarten ≥5 times per week/at least half of the times, (iii) residing 2-10 km from the workplace, (iv) residing <3 km from the kindergarten and the grocery shop, (v) possessing a smartphone, and (vi) being between 170-190 cm tall (due to the size of accessible bicycles). Exclusion criteria are: (i) engaging in PA sufficient to achieve the recommended level of PA (57), (ii) having bicycled more than once monthly during the last 12 months to each of the following destinations: workplace, kindergarten and grocery shop (i.e. >60 min monthly/>12 trips to each destination), and (iii) suffering from severe cardiovascular diseases or upper respiratory tract diseases. If necessary to achieve sufficient sample size, the target group will include parents of toddlers born in 2013 and 2015 recruited from kindergartens in the immediate area.

***Measurements***

*Questionnaire survey*

When signing up and providing consent electronically, parents will also supply relevant background information (e.g. gender, age, ethnicity, education, income and occupational status), and information allowing to determine eligibility for inclusion. Moreover, a web-based questionnaire will assess transportation habits, self-perceived health and HRQoL (58), and determinants for bicycling, e.g. intrinsic motivation (55), at baseline and post all intervention arms, i.e. in total 4 times.

*Bicycle use and total physical activity level*

Accelerometers are the most common devices for assessing PA objectively, yet to measure amount of cycling is especially challenging (59). In this regard, smartphones possess a great potential as they have GPS, accelerometers and gyroscope included, and provide a platform for PA monitoring without introducing participants to additional devices, burden or potential changes in behaviour. Besides, smartphone ownership is substantial; in 2015 95% of Norwegian adults aged 25-44 years reported to own a smartphone, when assessed in a representative sample (60). If combined with an appropriate smartphone application (app), data can be utilized and relevant measures of time in MVPA can be derived, in addition to cycling speed and –distance. Currently there are apps (e.g. Moves) that estimate and differentiate time spent on different modes of transportation such as bicycling, walking, running, and passive transportation (car/bus/train), yet there is no app measuring both type of transportation mode and level of MVPA combined. Such an appropriate smartphone app is currently being developed in cooperation with Dep. of Information and Communication Technology at University of Agder (UiA), and it will be validated prior use, in relation to established PA monitors in recordings of MVPA. The app will also include a diary-function, enabling registration of bicycle type (el/longtail/traditional) and trip purpose. When readily developed, the app will allow for cycling time (minutes), distance, speed and elevation gain (vertical ascent in meters) to be assessed, in addition to time spent in MVPA. If development of the app is delayed, the monitor SenseWear Armband Mini (SWA; BodyMedia, Pittsburgh, Pennsylvania, USA) will be used for estimating MVPA. In addition, seasonal variations will be accounted for and weather data (temperature, rainfall, snow, etc.) will be collected. Also, habitual modes of transportation and MVPA-level will be objectively measured for two weeks at study start, towards the end of each intervention arm, and at study completion, to explore potential changes in transportation habits and level of MVPA.

*Physiological parameters*

At baseline and post all intervention arms (in total 4 times), cardiorespiratory fitness will be measured performing treadmill walking/running to exhaustion, using a modified Balke-protocol according to Edvardsen et al. (61). VO_2_ max, minute ventilation (VE), and respiratory exchange ratio (RER) will be measured by mixing chamber, open-circuit indirect calorimetry. Heart rate (HR) will be registered every minute using the heart rate sensor Polar S610i (Polar Electro, Oy, Kempele, Finland). Time to exhaustion will be measured as minutes from start to test completion, i.e. reaching VO_2_ max. Criteria for acceptable VO_2_ max will be determined according to sex and age, as described by Edvardsen et al. (62). In order to obtain rapid and accurate estimates of total body composition, dual-energy X-ray absorptiometry (DXA) will be used, being considered a feasible reference method for measuring the three major components of the body, i.e. fat mass, lean mass and bone mineral mass (63). In addition, blood pressure will be measured with the monitor Microlife BP A100 (Widnau, Switzerland), and body weight and height (height only at baseline) will be measured to the nearest 0.1 kg and 0.5 cm, respectively, with participants (and their partners) wearing light clothes and no shoes. Oral and written instructions will be provided prior testing, and all measurements will be performed by the same test leaders and in the same order each time.

*Qualitative interviews*

Apart from the influence of environmental aspects (accessibility) on bicycling (45), we also want to address intrinsic motivation as one potentially relevant psychological determinant at the intrapersonal level, guided by Self-determination theory (SDT) as theoretical framework (55). Thus, participants in the intervention group (n=18) will be invited to participate in semi-structured qualitative interviews at study completion. Data collection method will be focus group interviews (n=6 in each group), chosen as an appropriate method for obtaining a nuanced exploration of the aspects of interest, based on group discussions and interactions between the participants (64). Main focus for the interviews will be exploration of participants’ experiences with usage of the different bicycle types, and further intrinsic motivation (56) for bicycling. One trained interviewer (with public health background) will lead the interviews, while an assistant will be present for observation.

***Quantitative and qualitative data analyses***

The statistical analyses for the quantitative data will be performed using the statistical software package IBM SPSS Statistics version 22.0 (IBM Corp., Somers, New York, USA). A two-sided p-value of <0.05 will be considered statistically significant. Descriptive analyses will be conducted and continuous variables will be presented as means and standard deviations (SD), categorical variables as proportions. The main analyses will be conducted according to Twisk and Proper (65), using data from the post-measures (i.e. at 12 months), adjusting for baseline measures.

A qualitative content analysis will be conducted from a hermeneutic perspective (66), Recordings of the qualitative interviews will be transcribed verbatim and read repeatedly to grasp the meaning of the data as a whole. Data will then be imported into the software analysis program NVivo 10 for further analysis. The next step will be to separate the text into meaning units; meaning the words, sentences or paragraphs containing aspects related to each other through their content and context. Following this process the meaning units will be classified into subcategories, by pooling data with similar characteristics together in a category defined by its content. Finally, the subcategories will be combined and categorized into main categories.

***Compliance with strategic documents and scientific relevance***

Study objectives of the present project are in accordance with the goal of the Paris Agreement adopted by 195 countries in December 2015, aiming to limit the increase in the global average temperature to well below 2 °C above pre-industrial levels (targeting 1.5 °C), as this would imply significantly reduced risks and impacts of climate change (24). This ambitious goal expresses the necessity of initiatives to be generated within all areas of society. Accordingly, a clearly stated aim for the Norwegian Government is to reduce man-made emissions of GHGs (67). Also, the National Transportation plan 2014-2023 states that cycling, together with walking and public transport, are main priorities (21), and the latest White Paper on public health, “Mastering and opportunities”, highlights active transportation in relation to making healthier choices easier (68). As cycling for transportation could increase total PA levels, mode shifts from motorized modes to bicycling is likely to contribute to increased compliance with the national (69) and Nordic (70) recommendations for PA. The present project could also support the explicit public health policy goal to reduce social inequalities in health (71), since cycling for transportation is cheaper than travelling by car. Although the prices of e-bikes compared with traditional bikes are relatively high, the prices are declining (33), and compared to cars e-bikes are affordable. This proposal is also very much in line with the strategic plan of University of Agder, Faculty of Health and Sport Sciences, where public health in general, and interventional research targeting physical activity more specifically, are among the priorities.

The present study will add knowledge to relevant and topical areas, i.e. issues related to public health and environmental sustainability, among parents of toddlers, representing a target group of greatest importance. There is a call for research on the influence of e-bikes on travel behavior and level of MVPA (34), together with increased understanding whether voluntary cycling with e-bikes could improve health. Moreover, to our knowledge no scientific studies have assessed potential effects of using a longtail bike on the currently selected parameters. If the present study reveals promising results, it should be replicated in a larger and more representative sample of kindergarten-parents, as well as in other important target groups (e.g. older adults). Generalizability to the adult population in general is likely to be high, yet this should also be tested in future studies before inferences can be made regarding potential effects of bicycling (e-bike/longtail/traditional) on the selected aspects under investigation. If findings are positive, inclusion in national public health policies should be considered.

***Likelihood of achievement***

Principal Investigator (PI) Elling Bere, professor in Public health science at UiA, has experience with research on active transportation, and from several large intervention studies; among them as WP leader for the evaluation of the Up4fun intervention within the large EU-financed ENERGY project. Bere is currently leading the research group Sustainable Lifestyles at UiA. Sveinung Berntsen, professor in Sport science at UiA, is also central in this project. He has been running several exercise intervention studies. At present, Berntsen leads the research group Physiological adaptation in sport, training and physical activity at UiA. There will also be a close collaboration with associate professor Saskia te Velde (UiA, VU University Medical Center Amsterdam), who is experienced with health promotion research and the usage of applications in research, and has been involved in several large intervention projects. She is experienced within research methodology and statistical analyses, and is currently hired at UiA in a 20% position. In addition, Liv Fegran, associate professor at UiA and a part time research supervisor at the pediatric unit in Sørlandet hospital, will be involved. Fegran is currently leading the research group Health promotion in a family perspective. Her research qualifications are in qualitative methods, both empirical primary studies (individual in-depth and focus group interviews) and review studies (metasynthesis). National and international collaborators will be represented by Lars Bo Anderssen (professor at Sogn og Fjordane University College and Norwegian School of Sports Sciences), who is a world recognized expert in the fields of active transportation, physical activity and health, Benedicte Deforche (professor at Ghent University, Belgium), holding great experience in research on active transportation and determinants for active transportation, and Aslak Fyhri (Norwegian Institute of Transport Economics), who is an expert on transportation research and -analyses, especially related to cycling (including e-bikes), through inclusion in an advisory board. Project coordinator will be Helga Birgit Bjørnarå (applicant and postdoc), submitting her PhD-thesis by the end of June 2016. In light of contemporary challenges related to public health and environmental sustainability, the PhD-thesis goes into certain diet and physical activity habits with potential health and sustainable properties, and further methodological considerations linked to these aspects.

Relevant laboratory facilities are available at UiA, together with a team holding competence on physiological measurements and testing. Masterstudents will be recruited for the physiological measurements and data collection. Also, cooperation with bicycle stores in Kristiansand will be established, in order to provide assistance to participants experiencing technical problems with the bicycles. Running costs are relatively low, and the time frame (described below) is reasonable. In total, likelihood of achievement should be high in the present project.

***Dissemination***

A total of 5 scientific papers, with topics conforming to the study objectives, will be published in peer-reviewed journals. We will deliver papers/posters at international conferences, and popular articles will be published in relevant Norwegian newspapers/other relevant media (e.g Aftenposten Viten, forskning.no). Also, potential end users will be invited to participate in the design of the study and dissemination of study results, to ensure its relevance. By including experienced stakeholders as active partners from an early stage, we will shift from an exclusively research (theoretical) focus to a more practical (real-world) focus. Hence, we plan to establish a reference group, inviting representatives from Nasjonalforeningen for folkehelsen, Kristiansand Municipality, Vest-Agder County, the Norwegian Directorate of Health, and transportsykkel.no, for participation. Additionally, two families representing the target group will be included in the reference group to ensure relevant user perspectives. Prior study start a “kick-off” meeting will be held, throughout the project there will be a dialogue, and there will be held a new meeting at study completion in order to involve the representatives in dissemination of the results. The close collaboration between the project group and the stakeholders makes an important platform for dissemination of knowledge directly through their channels, including political advocacy. A communication strategy for external communication will be developed, and other important stakeholders to achieve the key messages will be defined. Dissemination materials will also include websites (Nasjonalforeningen, municipalities, Vest-Agder County etc.) and social media accounts (Twitter, LinkedIn, and Facebook).

***Timeline***

| Time | Milestones |
| --- | --- |
| 1. jan 2017 | Study start |
| January 2017 | Writing application for ethical approval (REK/NSD) |
| Spring 2017 | Planning and recruitment of participants |
| Spring 2017 | Development of web-based questionnaire |
| Autumn 2017 | “Kick-off” meeting with the project group and user representatives |
| Autumn/winter 2017/18 | Baseline-measures  Intervention arm 1  Measures |
| Winter/spring 2017/18 | Intervention arm 2  Measures |
| Spring/summer 2018 | Intervention arm 3  Post-measures and qualitative interviews |
| Autumn 2018 | Summary and dissemination meeting with the project group and user representatives |
| Autumn 2018-autumn 2019 | Statistical analyses and writing of papers 1-5, dissemination of results |
| June 2018 and June 2019 | Presentation at the annual ISBNPA conference |
| 31. dec 2019 | Study completion |

**References**

1. WHO. Global recommendations on physical activity for health. 2010.

2. Hallal PC, Andersen LB, Bull FC, Guthold R, Haskell W, Ekelund U. Lancet; 380: 247-57, 2012.

3. Helsedirektoratet. Fysisk aktivitet og sedat tid blant voksne og eldre i Norge. Nasjonal kartlegging 2014-2015. Oslo. Helsedirektoratet. 2015.

4. Goenka S, Andersen LB. Lancet; S0140-6736(16)00348-2; 2016.

5. Lieberman LS. Appetite; 47: 3-9, 2006.

6. Lee I-M, Shiroma EJ, Lobelo F, Puska P, Blair SN, Katzmarzyk PT, et al. Lancet; 380: 219-29, 2012.

7. Blair SN. Br J Sports Med; 43:1-2, 2009.

8. de Nazelle A, Nieuwenhuijsen MJ, Antó JM, Brauer M, Briggs D, Braun-Fahrlander C, et al.Environ Int; 37:766-77, 2011.

9. Foley L, Panter J, Heinen E, Prins R, Ogilvie D. Int J Behav Nutr Phys Act; 12:161; 2015.

10. Hamer M, Chida Y. Prev Med; 46: 9-13, 2008.

11. Matthews CE, Jurj AL, Shu X-o, Li H-L, Yang G, Li Q, et al. Am J Epidemiol; 165: 1343-50, 2007.

12. Andersen LB, Schnohr P, Schroll M, Hein HO. Arch Intern Med; 160: 1621-1628, 2000.

13. Saunders LE, Green JM, Petticrew MP, Steinbach R, Roberts H. PLoS One; 8:e69912, 2013.

14. Laverty AA, Mindell JS, Webb EA, Millett C. Am J Prev Med; 45: 282-8, 2013.

15. Bassett Jr DR, Pucher J, Buehler R, Thompson DL, Crouter SE. J Phys Act Health; 5: 795-814, 2008.

16. Skreden M, Øverby NC, Sagedal LR, Vistad I, Torstveit MK, Lohne-Seiler H, et al. Int J Behav Nutr Phys Act; 13: 1-9, 2016

17. Andersen LB. Lancet Diabetes Endocrinol; 2016. DOI: <http://dx.doi.org/10.1016/S2213-8587(16)00077-2>

18. Flint E, Cummins S. Lancet Diabetes Endocrinol; 4: 420-35, 2016

19. Oldridge NB. Eur J Cardiovasc Prev Rehabil; 15: 130-9, 2008.

20. Sælensminde K. Positive helseeffekter av fysisk aktivitet En konkretisering av veien mot mer fullstendige samfunnsøkonomiske analyser. Oslo. Helsedirektoratet. 2008.

21. Samferdselsdepartementet. Meld. St. 26 (2012-2013). Nasjonal transportplan (2014-2023). Oslo.Samferdselsdepartementet. 2012.

22. Woodcock J, Edwards P, Tonne C, Armstrong BG, Ashiru O, Banister D, et al. Lancet; 374: 1930-43, 2009.

23. Abagnale C, Cardone M, Iodice P, Strano S, Terzo M, Vorraro G. Environ Impact Assess; 53: 1-7, 2015.

24. COP21. United nations conference on climate change Paris 2015. Available from: <http://www.cop21.gouv.fr/en>. 2015

25. Vegdirektoratet. Nasjonal sykkelstrategi 2014-2023- Sats på sykkel! Oslo. Vegdirektoratet. 2012.

26. Craigie AM, Lake AA, Kelly SA, Adamson AJ, Mathers JC. Maturitas; 70: 266-84, 2011.

27. Biddle SJ, Pearson N, Ross GM, Braithwaite R. Prev Med; 51: 345-51, 2010.

28. The NS, Suchindran C, North KE, Popkin BM, Gordon-Larsen P. JAMA; 304: 2042-7, 2010.

29. Starc G, Strel J. Public Health Nutr; 14: 49-55, 2011.

30. Sallis JF, Prochaska JJ, Taylor WC. Med Sci Sports Exerc; 32: 963-75, 2000.

31. Trost SG, Owen N, Bauman AE, Sallis JF, Brown W. Med Sci Sports Exerc; 34:1996-2001, 2002.

32. Bauman AE, Reis RS, Sallis JF, Wells JC, Loos RJF, Martin BW. Lancet; 380: 258-71, 2012.

33. Weiss M, Dekker P, Moro A, Scholz H, Patel MK. Transportation Research Part D: Transport and Environment.; 41:348-66, 2015.

34. Fishman E, Cherry C. Transport.Rev; 36: 72-91, 2016.

35. Karlsen C, Knudsen O; http://www.osloby.no/nyheter/sykkelpatruljen/Slik-klarer-smabarnspappaen-seg-fint-uten-bil-8363161.html. 2016.

36. Johnson M, Rose G. J Transp Health; 2: 276-83, 2015.

37. Fyhri A, Fearnley N; Transportation Research Part D: Transport and Environment; 36: 45-52, 2015.

38. Cherry CR, Yang H, Jones LR, He M. Transport Policy; 45: 127-35, 2016.

39. Astegiano P, Tampère CM, Beckx C. Transportation Research Procedia; 10: 393-402, 2015.

40. Shephard RJ. Curr Cardiovasc Risk Rep; 6: 299-306, 2012.

41. Simons M, Van Es E, Hendriksen I. Med Sci Sports Exerc; 41: 2097-102, 2009.

42. Gojanovic B, Welker J, Iglesias K, Daucourt C, Gremion G. Med Sci Sports Exerc; 43: 2204-10, 2011.

43. Sperlich B, Zinner C, Hébert-Losier K, Born D-P, Holmberg H-C. Eur J Appl Physiol; 112: 4015-25, 2012.

44. De Geus B, Kempenaers F, Lataire P, Meeusen R. Eur J Sports Sci; 13: 290-4, 2013.

45. Sallis JF, Cervero RB, Ascher W, Henderson KA, Kraft MK, Kerr J. Annu Rev Public Health. 2006;27:297-322.

46. Cairns S, Behrendt F, Raffo D, Harmer C. J Transp Health; 2: S17, 2015.

47. Fishman E. Transport Rev; 36: 92-113, 2016.

48. Langford B, Cherry C, Yoon T, Worley S, Smith D. J Transport Research Board; 2387:120-8, 2013.

49. Fishman E, Washington S, Haworth N. Transportation Research Part D: Transport and Environment; 31: 13-20, 2014.

50. Fishman E, Washington S, Haworth N. J Transp Health; 2: 135-42, 2015.

51. Woodcock J, Tainio M, Cheshire J, O’Brien O, Goodman A. BMJ; 348:g4252014, 2014.

52. Wen LM, Kite J, Rissel C. BMC Public Health; 10: 50, 2010.

53. Bopp M, Child S, Campbell M. Women & health; 54: 212-31, 2014.

54. Yang L, Hipp JA, Adlakha D, Marx CM, Tabak RG, Brownson RC. J Transp Health;2: 212-8, 2015.

55. Ryan RM, Deci EL. Am Psychol; 55: 68-78, 2000.

56. Deci EL, Ryan RM. Psychological inquiry; 11: 227-68, 2000.

57. Haskell WL, Lee I-M, Pate RR, Powell KE, Blair SN, Franklin BA, et al. Circulation; 116: 1081, 2007.

58. Moons P, Budts W, De Geest S. Int J Nurs Stud; 43: 891-901, 2006.

59. Matthews CE. Med Sci Sports Exerc; 37:S512-S522, 2005.

60. Medie Norge; Andel som har smarttelefon. Available from: http://www.medienorge.uib.no/statistikk/aspekt/tilgang-og-bruk/388.2016

61. Edvardsen E, Scient C, Hansen BH, Holme IM, Dyrstad SM, Anderssen SA. Chest; 144: 241-8, 2013.

62. Edvardsen E, Hem E, Anderssen SA. PLoS One.; 9: e85276, 2014.

63. St-Onge M-P, Wang J, Shen W, Wang Z, Allison DB, Heshka S, et al. The Journals of Gerontology Series A: Biological Sciences and Medical Sciences; 59:B796-B800, 2004.

64. Malterud K. Fokusgrupper som forskningsmetode for medisin og helsefag. Oslo. Universitetsforlaget. 2012.

65. Twisk J, Proper K. J Clin Epidemiol; 57: 223-8, 2004.

66. Kvale S, Brinkmann S. Det kvalitative forskningsintervju. Oslo, Gyldendal akademisk. 2015.

67. Miljøverndepartementet. Meld. St. 34 (2006-2007). Norsk klimapolitikk. Oslo. Miljøverndepartementet. 2007.

68. Helse-og omsorgsdepartementet. Meld. St. 19 (2014-2015). Folkehelsemeldingen: Mestring og muligheter. Oslo. Helse-og omsorgsdepartementet. 2015.

69. Helsedirektoratet. Anbefalinger om kosthold, ernæring og fysisk aktivitet. Oslo. Helsedirektoratet. 2014.

70. Nordic Council of Ministers. Nordic Nutrition Recommendations 2012- integrating nutrition and physical activity. Copenhagen. Nordic Council of Ministers. 2014.

71. Sosial- og helsedirektoratet. Gradientutfordringen. Sosial-og helsedirektoratets handlingsplan mot sosiale ulikheter i helse. Oslo. Sosial-og helsedirektoratet. 2005.
